# Supplementary material for: Oxynitrides enabled photoelectrochemical water splitting with over 3,000 hrs stable operation in practical two-electrode configuration
Source: Nat Commun. 2023 Apr 11;14:2047. doi: 10.1038/s41467-023-37754-9 (PMC10090041; doi:10.1038/s41467-023-37754-9)
Supplement: Supplementary file 1 — Supplementary Information [file 41467_2023_37754_MOESM1_ESM.pdf]

Supplementary Information for

Oxynitrides: Enabling Photoelectrochemical Water Splitting with over 3,000 hrs Stable Operation in Practical Two-Electrode Configuration

Yixin Xiao[1#], Xianghua Kong[2#], Srinivas Vanka[1#], Wan Jae Dong[1#], Guosong Zeng[3], Zhengwei Ye[1], Kai Sun[4], Ishtiaque Ahmed Navid[1], Baowen Zhou[1], Francesca M. Toma[3], Hong Guo[2], and Zetian Mi[1*]*

[1]Department of Electrical Engineering and Computer Science, University of Michigan, Ann Arbor, 1301 Beal Avenue, Ann Arbor, MI 48109, USA

[2]Department of Physics, McGill University, 3600 University Street, Montreal, Quebec H3A 2T8, Canada

[3]Lawrence Berkeley National Laboratory, Chemical Sciences Division, 1 Cyclotron Road, Berkeley, CA 94720, USA

[4]Department of Materials Science and Engineering, University of Michigan, 2300 Hayward Street, Ann Arbor, MI 48109, USA

[*]*Corresponding authors email addresses:* ztmi@umich.edu *(Z.M.),* hong.guo@mcgill.ca *(H.G.)*

[#]*Equal contribution by authors*

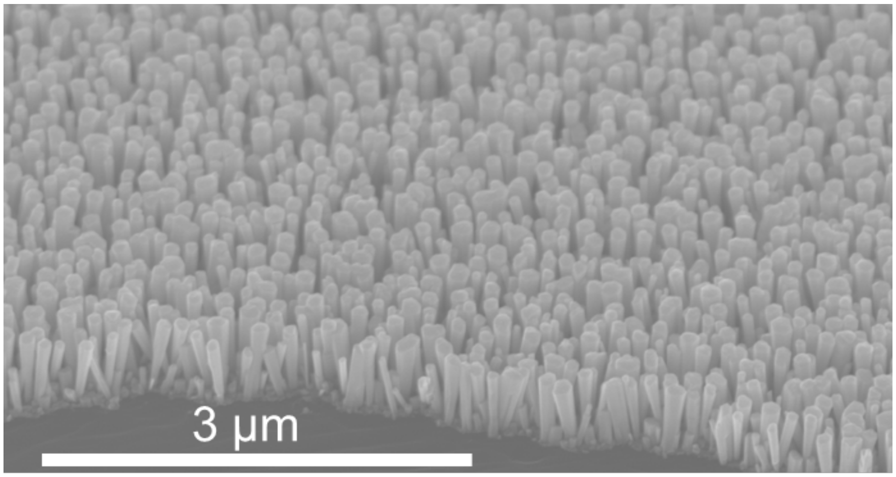

Figure S1. Scanning electron micrograph of the as-grown n^+-GaN nanowires/Si photocathode.

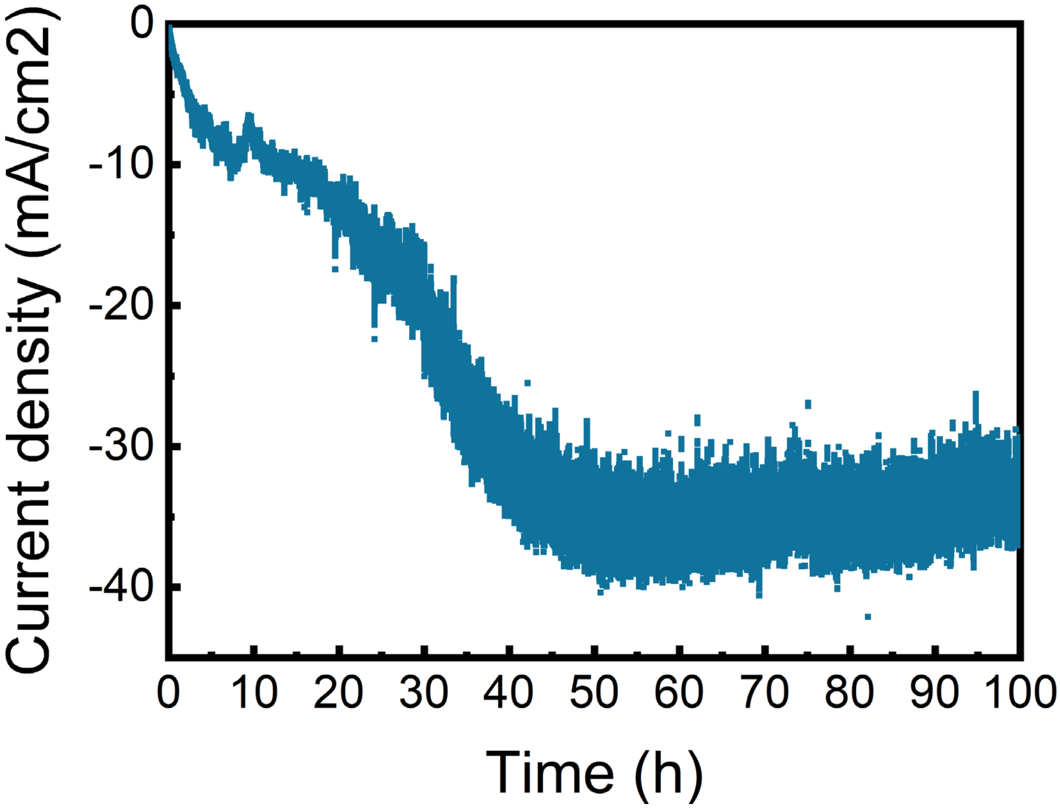

Figure S2. Chronoamperometry (CA) curve of the GaN NW/Si photocathode at -0.4 V *vs.* reversible hydrogen electrode under AM 1.5G one-sun illumination in 0.5 M H_2SO_4.

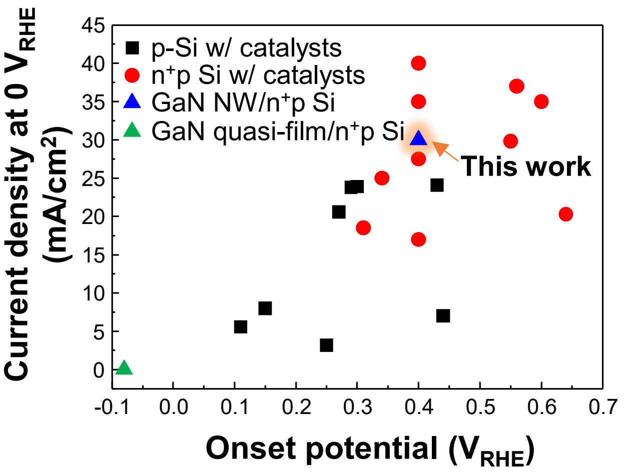

Figure S3. Performance comparison of Si photocathodes for H_2 evolution reaction. The Y-axis indicates the photocurrent density at 0 V vs. reversible hydrogen electrode (V_{RHE}) and the X-axis indicates the onset potential (V_{on}). n^+-p Si photocathodes with catalysts (red circles) show better performance than *p*-type Si photocathodes with catalysts (black squares). Notably, GaN NW/Si (this work, highlighted blue triangle) shows a significant advancement from the previous GaN quasi-film/Si photocathode (green triangle)[1] due to the beneficial nanowire morphology of GaN.

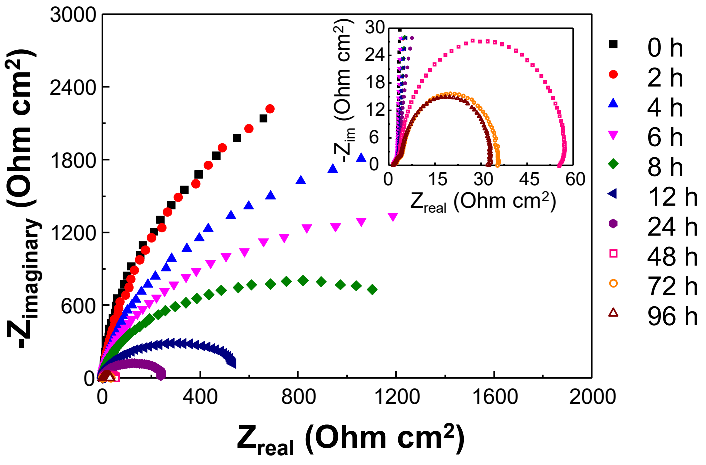

Figure S4. Photoelectrochemical Nyquist impedance plots of GaN NW/Si after the CA test for each period time (0 – 96 h). The measurements were conducted at -0.4 V_{RHE} under AM 1.5G 1 sun light illumination. Inset shows a magnified graph of the low resistance region.

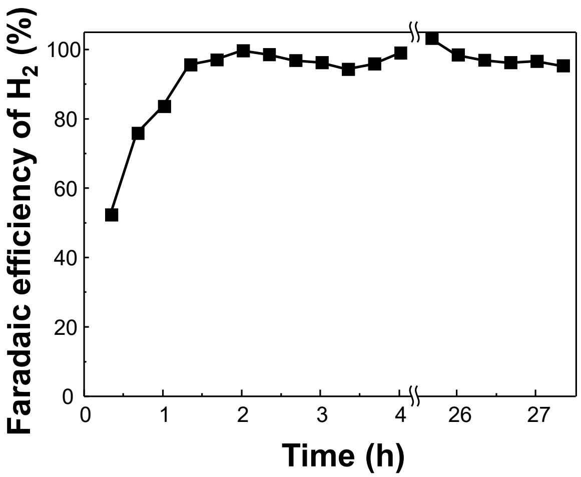

Figure S5. Faradaic efficiency of H_2 measured at -0.4 V_{RHE} for initial 4 h and after CA test (25 h).

The measurements were taken at -0.4 V_{RHE} under AM 1.5G 1 sun light illumination.

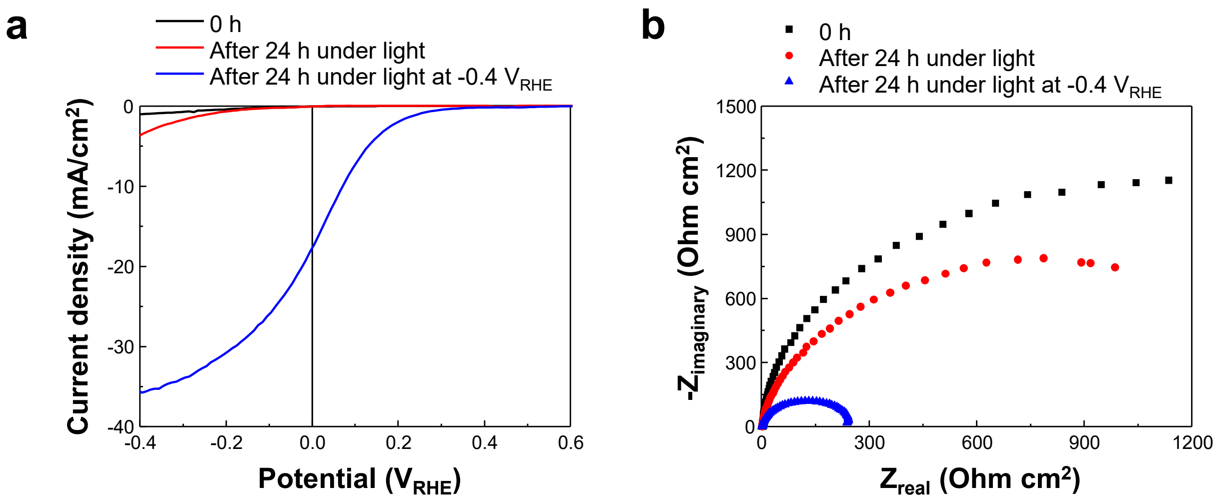

Figure S6. (a) Linear sweep voltammetry curves and (b) Nyquist plots of GaN NW/Si photocathodes; pristine (0 h, black), under one-sun illumination at open-circuit potential (24 h, red), and under one-sun illumination at -0.4 V_{RHE} (24 h, blue). Both light illumination and cathodic potential are required for the self-improvement of GaN NW/Si photocathode.

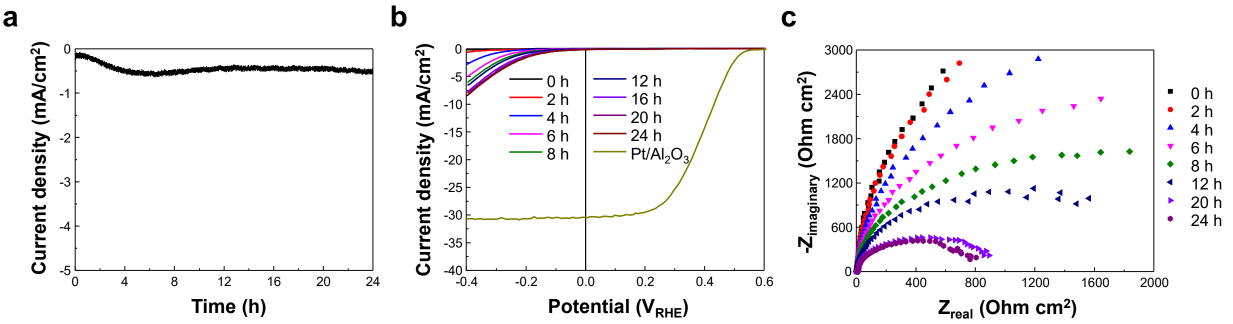

Figure S7. (a) CA curve, (b) LSV curves, and (c) Nyquist impedance plots of Al_2O_3 (2 nm)-passivated GaN NW/Si photocathodes. There was very little increase in photocurrent density and decrease in charge transfer resistance after 24 hours of reaction at -0.4 V_{RHE} under one-sun illumination. Even after 24 hours of chronoamperometry, the negative V_{on} (< 0 V_{RHE}) and the small photocurrent density (< 10 mA/cm^2 at -0.4 V_{RHE}) clearly demonstrate that the performance is not as good as GaN NW/Si (V_{on} > 0 V_{RHE} and the photocurrent density > 30 mA/cm^2 at -0.4 V_{RHE}). Panel (b) also shows that, after a routine photodeposition of Pt cocatalysts, the activity of the Al_2O_3 passivated photocathode is comparable to one without Al_2O_3 passivation (after Pt deposition).

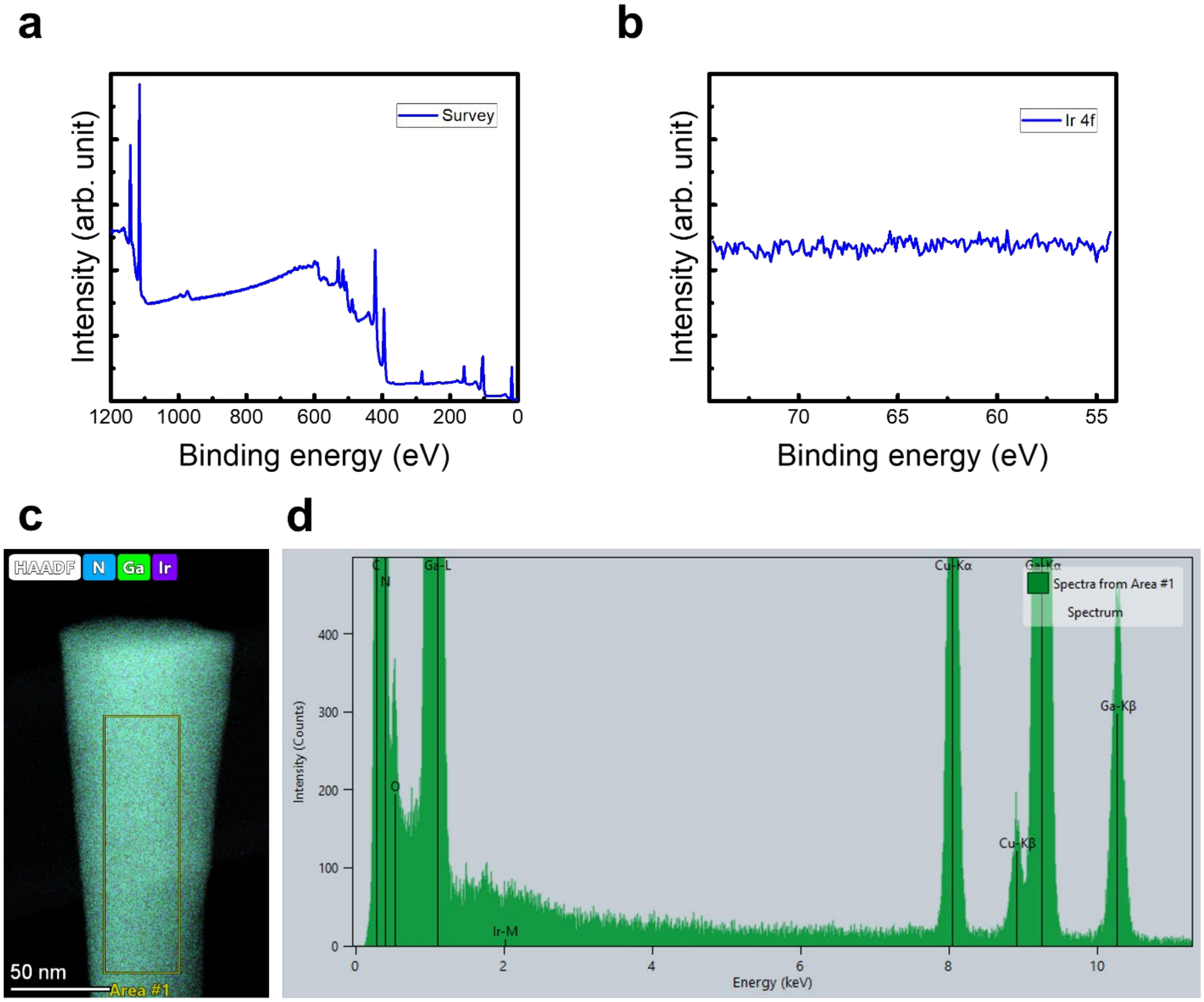

Figure S8. (a) Survey spectrum of X-ray photoelectron spectroscopy (XPS) of the GaN NW/Si photocathode after 48 hours of chronoamperometry at -0.4 V_{RHE} under one-sun illumination. (b) XPS of the GaN NW/Si photocathode shows no Ir after 48 hours of chronoamperometry at -0.4 V_{RHE} under one-sun illumination. (c) Region of interest for the Transmission electron microscopy (TEM) energy dispersive X-ray spectroscopy (EDS) spectrum is given by the box with yellow outlines and label "Area #1". (d) The TEM EDS spectrum in the region of interest shows no Ir after reaction with conditions given above.

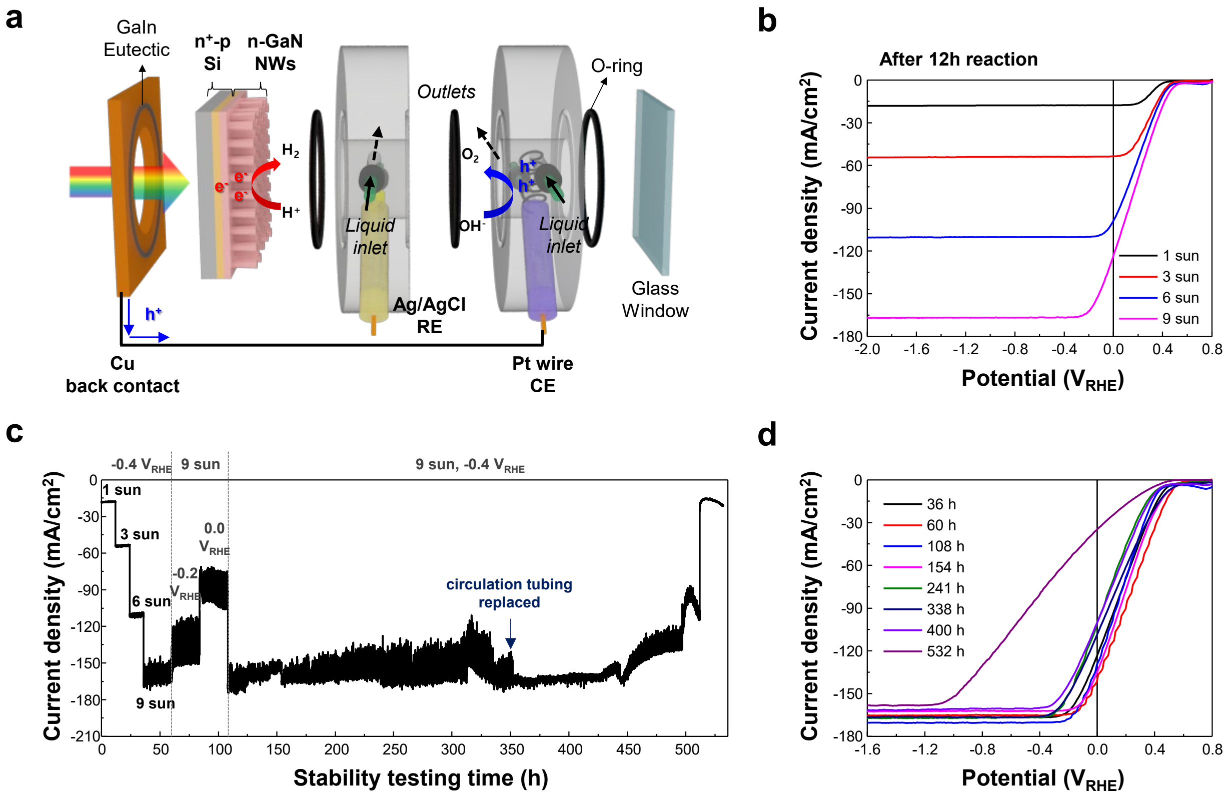

Figure S9. (a) Schematic illustration of the flow cell for concentrated solar light photoelectrochemical H_2 evolution reaction. Simulated solar spectrum is irradiated on the backside of Si wafer and reaction takes place on the front side of GaN NWs. Electrolyte was continuously flowed with a rate of 28 ml/min. (b) LSV curves of CA-tested GaN NW/Si for 12 h under nine-sun illumination. The saturated photocurrent densities linearly correlate to the light intensity. (c) CA curves measured at different light intensities and potentials. (d) LSV curves of GaN NW/Si after the CA test for each period time (0 – 532 h) under the concentrated solar light (9 sun).

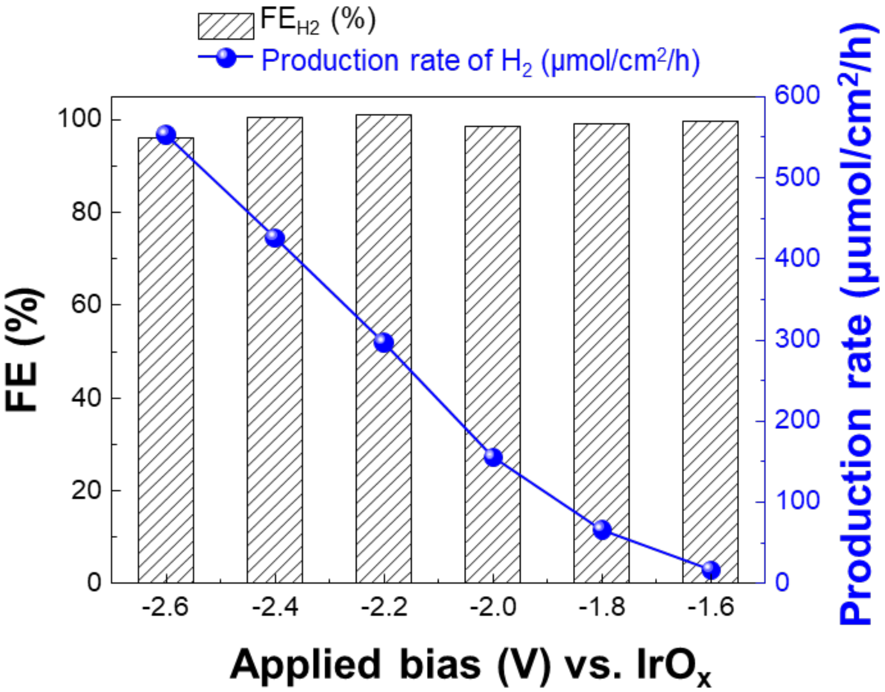

Figure S10. Faradaic efficiency of the GaN NW/Si photoelectrode at different biases after 24 hr of chronoamperometry at -2 V *vs.* IrO_x under one-sun illumination.

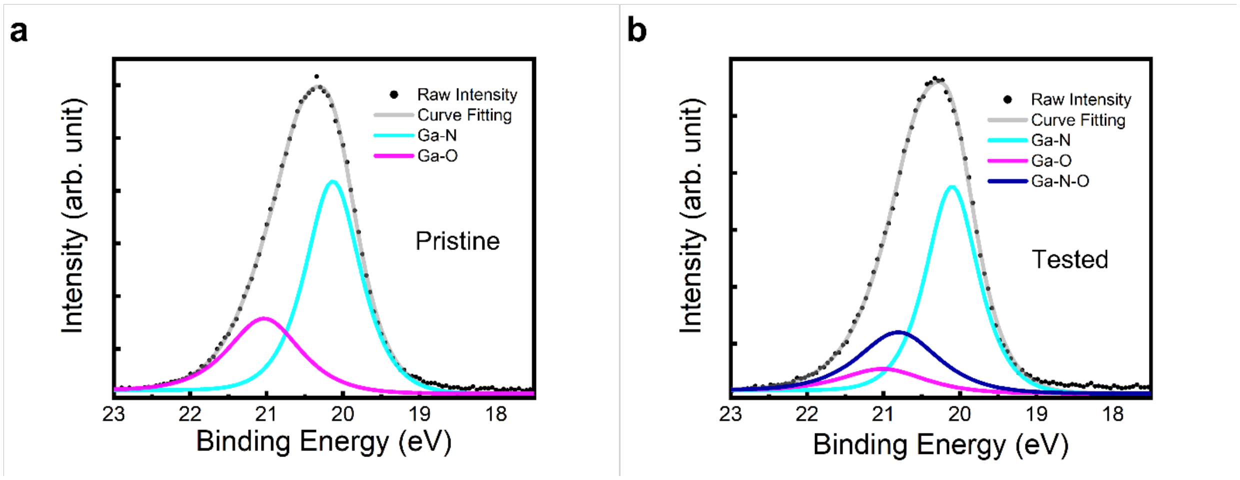

Figure S11. XPS measurements for *Ga 3d* of pristine (0 h) sample (a) showing deconvoluted peaks at 20.2 eV and 21 eV for Ga-N (cyan curve) and Ga-O (magenta curve), respectively. The curve fitting of the raw XPS data is the gray curve. *Ga 3d* peaks of the tested (after 10 h) sample (b) show an additional deconvoluted peak at 20.8 eV corresponding to gallium oxynitride species (navy blue curve).

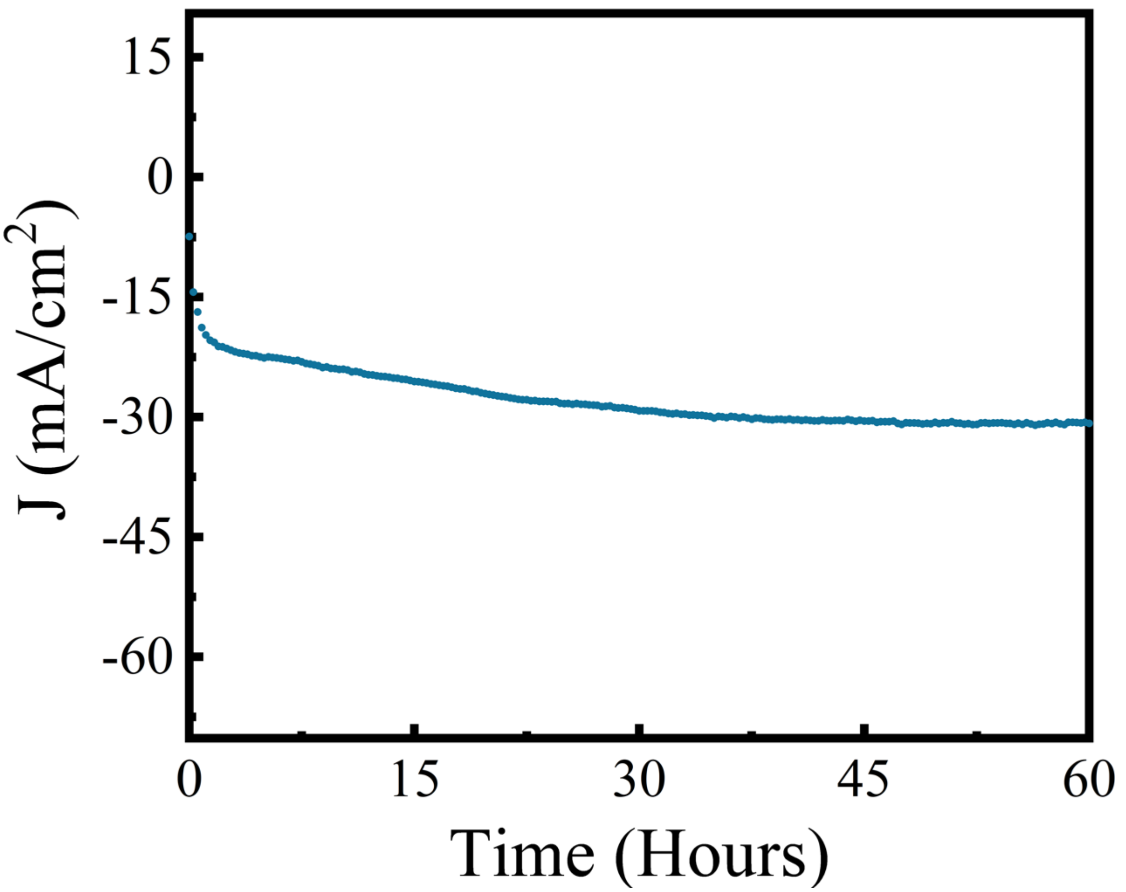

Figure S12. Chronoamperometry results for the first 60 h of the stability experiments in the two-electrode configuration under AM 1.5G one-sun illumination 0.5 M H_2SO_4 in with 0.2 mM Triton X-100 at -2.3 V *vs.* IrO_x. The photocurrent density reached a saturated value of ~ 30 mA/cm^2 after ~ 40 h.

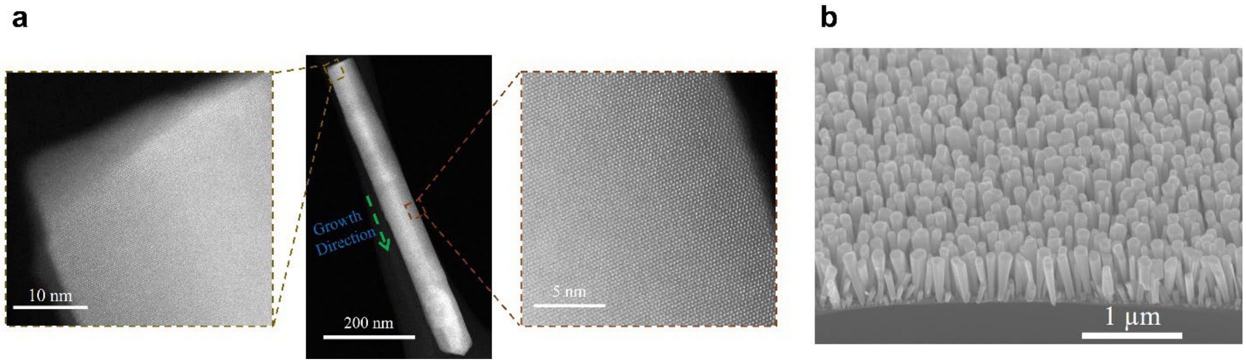

Figure S13. (a) Scanning transmission electron microscopy (STEM) image of an n^+-GaN nanowire at the end of the 3000 h chronoamperometry. No apparent change in nanowire morphology was observed (length ~ 600 nm and diameter ~ 100 nm). Brown boxes represent atomic resolution High-angle annular dark-field STEM images of highly crystalline GaN nanostructure across the wire. (b) Scanning electron micrograph of the n^+-GaN nanowire on Si photocathode at the end of the 3000 h chronoamperometry. No apparent change in morphology was observed.

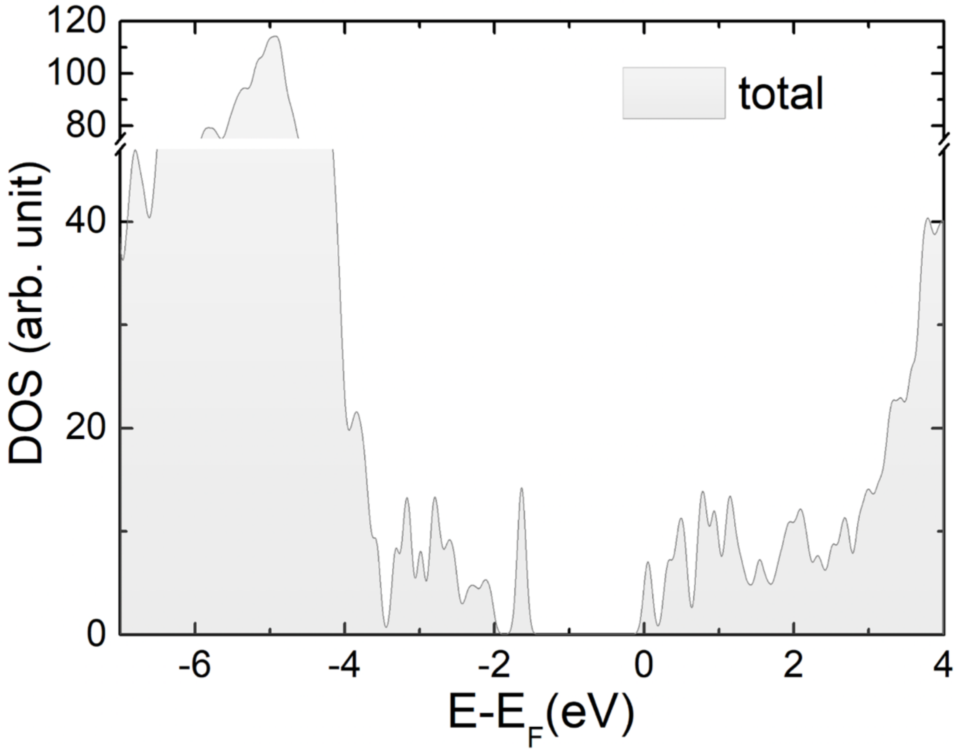

Figure S14. Density of states (DOS) of N-rich GaN *m*-plane before oxidation.

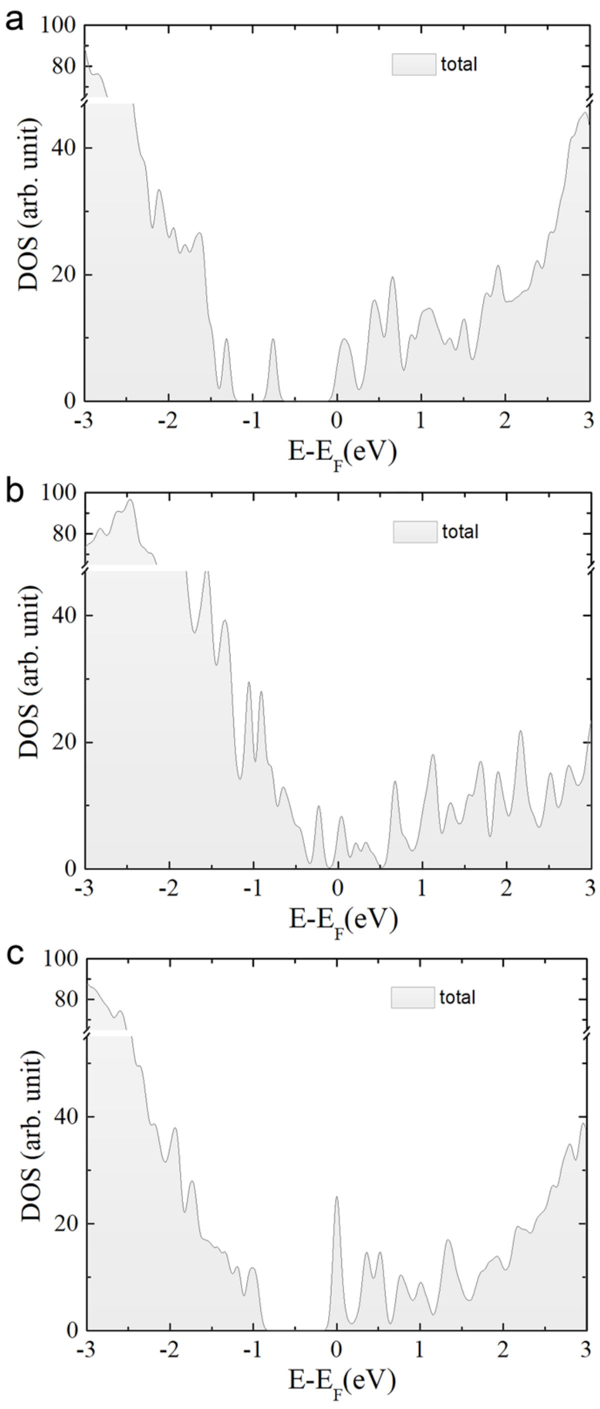

Figure S15. Density of states (DOS) of metal oxynitride species featured GaN m-plane. (a) – (c) DOS of the first, third and fourth configuration plotted in Figure 5a. Their formation energies are -4.07 eV, -3.95 eV and -2.66 eV, respectively. DOS of the first, third and fourth configurations was calculated with optB86-vdW.

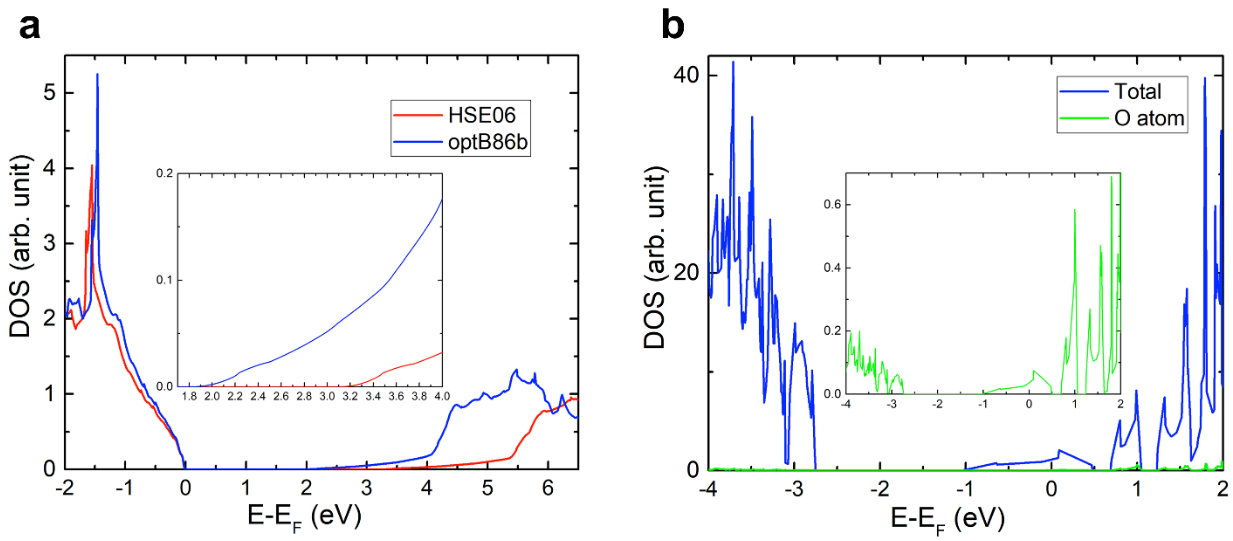

Figure S16. Density of states (DOS) of (a) pristine and (b) metal oxynitride featured bulk wz-GaN.

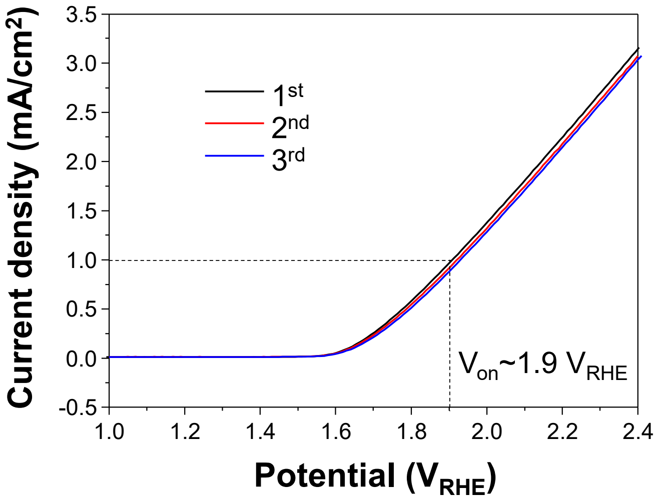

Figure S17. Linear sweep voltammetry curves of the IrO_x counter electrode measured in 0.5 M H_2SO_4. Three individual measurements were performed and the averaged onset potential at 1 mA/cm^2 was ~1.9 V_{RHE}.

Table S1. Comparison between the stability of state-of-the-art photoelectrodes in two- and three-electrode configurations and the GaN nanowires/Si photocathode presented in this work.

Material	Photoelectrode	Stability (Hours)		Ref.
		Two-Electrode	Three-Electrode	
PtRu-GaInP/AlInP/GaInP-GaInP/GaInAs	Photocathode	0.4	10	2
Rh-TiO_2-AlInP/GaInP-/GaInAs/GaAs-RuO_x	Photocathode	0.5	30	3
Rh/AlInP-GaInP/GaInAs-RuO_2	Photocathode	0.1	40	4
InGaP/GaAs double junction	Photoanode	15	150	5
3J *a*-Si SC with Co/NiMoZn	Photoanode	0.2	0.5	6
Co-Ci dual doped $BiVO_4$/1J perovskite solar cell	Photoanode	12	<12	7
$BiVO_4$‖Fe_2O_3- 2J Si	Photoanode	8	8	8
Fe_2O_3/perovskite tandem cell	Photoanode	0.95	8	9
Pt/GaN/3J $GaInP_2$/GaAs/Ge	Photocathode	60	80	10
Ni-TiO_2/GaInP/GaAs	Photoanode	40	NA	11
Co-Pi Mn doped Fe_2O_3-1J perovskite solar cell	Photoanode	8	NA	12
Co-Pi Gradient W: $BiVO_4$/2J a:Si solar cell	Photoanode	1	NA	13
Pt/$GaInP_2$/GaAs	Photocathode	10	NA	14
WO_3/$BiVO_4$-DSSC	Photoanode	2	NA	15
WO_3/$BiVO_4$/GaAs/InGaAsP	PV-PEC	1	NA	16
Pt-TiO_2-3J-Solar cell	Photocathode	2	NA	17
CdS/CdSe/DSSC	Photoanode	2	NA	18
CoPi/$BiVO_4$/p^+n Si	Photoanode	1	NA	19
Fe_2O_3/DSC tandem cell	Photoanode	8	NA	20
Pt/1J InGaN	Photocathode	300	NA	21
**Ga(O)N nanowires/Si**	**Photocathode**	**3000**	**NA**	**This work**

Table S2. Performance comparison of reported Si photocathodes for H_2 evolution reaction.

Photocathode	Passivation	Cocatalyst	Electrolyte	Onset potential (V_{RHE})	Current density at 0.0 V_{RHE} (mA/cm^2)	Ref.
p-Si	-	Ni/Fe:In_2S_3	0.5 M Na_2SO_3	0.44	7.0	[22]
p-Si	-	$Ni(TEOA)_2Cl_2$	0.5 M H_2SO_4	0.11	5.6	[23]
Mesoporous p-Si	-	Co-P	0.5 M H_2SO_4	0.3	23.9	[24]
p-Si microwires	-	CoS_2	0.5 M H_2SO_4	0.25	3.2	[25]
p-Si	-	Mo_3S_4	1 M $HClO_4$	0.15	8	[26]
p-Si	-	MoS_xCl_y	0.5 M H_2SO_4	0.27	20.6	[27]
p-Si	TiO_2	Pt	1 M $HClO_4$	0.48	30	[28]
p-Si	TiO_2	MoS_2/Rh-P	0.5 M H_2SO_4	0.43	24.1	[29]
p-Si pyramid	TiO_2	$MoS_{1.75}P_{0.25}$	0.5 M H_2SO_4	0.29	23.8	[30]
n^+p Si	-	MoS_2-Mo_3S_{13}	0.5 M H_2SO_4	0.4	17	[31]
n^+p Si	Mo_xSi/SiO_2	MoS_2	0.5 M H_2SO_4	0.31	18.5	[32]
n^+p Si microwires	NiSi	NiMo	1 M KOH	0.55	29.8	[33]
n^+p Si	TiO_2	Pt	0.5 M H_2SO_4	0.64	20.3	[34]
n^+p Si	Al_2O_3	MoS_2	1 M $HClO_4$	0.4	35	[35]
n^+p Si pyramid	TiO_2	Pt	1 M $HClO_4$	0.6	35	[36]
n^+p Si pyramid	-	$MoSe_2$	1 M $HClO_4$	0.4	27.5	[37]
n^+p Si	GaN nanowires	Pt	0.5 M H_2SO_4	0.56	37	[38]
n^+p Si	GaN nanowires	MoS_2	0.5 M H_2SO_4	0.4	40	[39]
n^+p Si	GaN quasi-film	Pt	0.5 M H_2SO_4	0.34	25	[1]
n^+p Si	GaN quasi-film	-	0.5 M H_2SO_4	-0.08	0	
n^+p Si	GaN nanowires	-	0.5 M H_2SO_4	0.4	30	**This work**

Table S3. Summary table showing average V_{on} and average J_{ph} during different cycles of the long-term stability experiments.

Start of cycle (h)	End of cycle (h)	Average V_{on} (V *vs.* IrO_x)	Average $\|J_{ph}\|$ at -2.3 V *vs.* IrO_x (mA/cm^2)
0	100	-1.5	28.3
100	200	-1.35	29
200	300	-1.4	29.1
300	400	-1.42	29.5
400	500	-1.4	29
500	600	-1.38	30.4
600	700	-1.4	30.2
700	800	-1.43	30.8
800	900	-1.4	30.6
900	1000	-1.39	30
1000	1100	-1.41	29.8
1100	1200	-1.4	29.4
1200	1300	-1.38	30.8
1300	1400	-1.35	30.1
1400	1500	-1.35	30.4
1500	1600	-1.38	30.5
1600	1700	-1.4	30.2
1700	1800	-1.41	29.9
1800	1900	-1.38	30.5
1900	2000	-1.43	30.1
2000	2100	-1.4	30
2100	2200	-1.42	30.7
2200	2300	-1.38	30.1
2300	2400	-1.39	29.5
2400	2500	-1.43	29.4
2500	2600	-1.4	30.4
2600	2700	-1.4	30.2
2700	2800	-1.39	30.6
2800	2900	-1.4	29.7
2900	3000	-1.42	28

Table S4. Summary of state-of-the-art metal oxynitrides and their stability under solar water splitting conditions.

Material	Electrolyte	Stability	pH	Ref.
TaON	Na_2SO_4	<3 h	8	[21]
TiON	0.1 M KOH	NA	14	[22]
$(Ga_{1-x}Zn_x)(N_{1-x}O_x)$	$H_2SO_4 + NaNO_2$	35	4.5	[23]
$LaTiO_2N$	10 mM $AgNO_3$	40	8	[23,24]
$LaMg_xTa_{1-x}O_{1+3x}N_{2-3x}$	H_2O	48	8	[25]
GaON	0.5M Na_2SO_4	NA	7	[26]
**Ga(O)N/Si**	**0.5M H_2SO_4**	**3000**	**0.16**	**This work**

Table S5. ICP-MS measurements of Ga element at different durations during the 3,000 h stability test.

Time (Hours)	Ga (nmol)
500-524	12.21
1000-1024	11.18
2000-2024	11.18
2980-3000	11.18

Table S6. Formation energy for GaON species on N-rich and pristine GaN *m*-plane for the second configuration in Figure 5(a). In general, metal-O bonds are stronger than metal-N bonds. This is because oxygen is more electronegative than nitrogen, meaning that it attracts electrons more strongly. As a result, the metal-O bond is more polarized and the oxygen atom carries a partial negative charge, while the metal carries a partial positive charge. This polarization leads to a stronger bond. Additionally, oxygen is a smaller atom than nitrogen, so the metal-O bond is also shorter and stronger due to the stronger overlap of atomic orbitals.

	Formation energy (eV)	
	O-N replacement	O adsorption
N-rich *m*-plane	-4.29	-4.19
m-plane	-3.14	-1.13

References

1 Zeng, G. *et al.* Development of a photoelectrochemically self-improving Si/GaN photocathode for efficient and durable H2 production. *Nature Materials* **20**, 1130-1135 (2021).

2 Young, J. L. *et al.* Direct solar-to-hydrogen conversion via inverted metamorphic multi-junction semiconductor architectures. *Nature Energy* **2**, 17028, doi:10.1038/nenergy.2017.28 https://www.nature.com/articles/nenergy201728#supplementary-information (2017).

3 Cheng, W.-H. *et al.* Monolithic Photoelectrochemical Device for Direct Water Splitting with 19% Efficiency. *ACS Energy Letters* **3**, 1795-1800, doi:10.1021/acsenergylett.8b00920 (2018).

4 May, M. M., Lewerenz, H. J., Lackner, D., Dimroth, F. & Hannappel, T. Efficient direct solar-to-hydrogen conversion by in situ interface transformation of a tandem structure. *Nat Commun* **6**, 8286, doi:10.1038/ncomms9286 (2015).

5 Varadhan, P., Fu, H. C., Kao, Y. C., Horng, R. H. & He, J. H. An efficient and stable photoelectrochemical system with 9% solar-to-hydrogen conversion efficiency via InGaP/GaAs double junction. *Nat Commun* **10**, 5282, doi:10.1038/s41467-019-12977-x (2019).

6 Reece, S. Y. *et al.* Wireless Solar Water Splitting Using Silicon-Based Semiconductors and Earth-Abundant Catalysts. *Science* **334**, 645-648, doi:10.1126/science.1209816 (2011).

7 Kim, J. H. *et al.* Wireless Solar Water Splitting Device with Robust Cobalt-Catalyzed, Dual-Doped BiVO4 Photoanode and Perovskite Solar Cell in Tandem: A Dual Absorber Artificial Leaf. *ACS Nano* **9**, 11820-11829, doi:10.1021/acsnano.5b03859 (2015).

8 Kim, J. H. *et al.* Hetero-type dual photoanodes for unbiased solar water splitting with extended light harvesting. *Nat Commun* **7**, 13380, doi:10.1038/ncomms13380 (2016).

9 Morales-Guio, C. G. *et al.* An Optically Transparent Iron Nickel Oxide Catalyst for Solar Water Splitting. *J Am Chem Soc* **137**, 9927-9936, doi:10.1021/jacs.5b05544 (2015).

10 Wang, Y., Schwartz, J., Gim, J., Hovden, R. & Mi, Z. Stable Unassisted Solar Water Splitting on Semiconductor Photocathodes Protected by Multifunctional GaN Nanostructures. *ACS Energy Letters* **4**, 1541-1548, doi:10.1021/acsenergylett.9b00549 (2019).

11 Verlage, E. *et al.* A monolithically integrated, intrinsically safe, 10% efficient, solar-driven water-splitting system based on active, stable earth-abundant electrocatalysts in conjunction with tandem III–V light absorbers protected by amorphous TiO2 films. *Energy & Environmental Science* **8**, 3166-3172, doi:10.1039/c5ee01786f (2015).

12 Gurudayal *et al.* Perovskite-Hematite Tandem Cells for Efficient Overall Solar Driven Water Splitting. *Nano Lett* **15**, 3833-3839, doi:10.1021/acs.nanolett.5b00616 (2015).

13 Abdi, F. F. *et al.* Efficient solar water splitting by enhanced charge separation in a bismuth vanadate-silicon tandem photoelectrode. *Nat Commun* **4**, 2195, doi:10.1038/ncomms3195 (2013).

14 Khaselev, O. & Turner, J. A. A monolithic photovoltaic-photoelectrochemical device for hydrogen production via water splitting. *Science* **280**, 425-427, doi:DOI 10.1126/science.280.5362.425 (1998).

15 Shi, X. *et al.* Unassisted photoelectrochemical water splitting beyond 5.7% solar-to-hydrogen conversion efficiency by a wireless monolithic photoanode/dye-sensitised solar cell tandem device. *Nano Energy* **13**, 182-191, doi:10.1016/j.nanoen.2015.02.018 (2015).

16 Kosar, S. *et al.* Tandem photovoltaic–photoelectrochemical GaAs/InGaAsP–WO3/BiVO4device for solar hydrogen generation. *Japanese Journal of Applied Physics* **55**, 04ES01, doi:10.7567/jjap.55.04es01 (2016).

17 Okamoto, S., Deguchi, M. & Yotsuhashi, S. Modulated III–V Triple-Junction Solar Cell Wireless Device for Efficient Water Splitting. *The Journal of Physical Chemistry C* **121**, 1393-1398, doi:10.1021/acs.jpcc.6b07991 (2017).

18 Shin, K. & Park, J. H. Highly Transparent Dual-Sensitized Titanium Dioxide Nanotube Arrays for Spontaneous Solar Water Splitting Tandem Configuration. *ACS Applied Materials & Interfaces* **7**, 18429-18434, doi:10.1021/acsami.5b04521 (2015).

19 Chakthranont, P., Hellstern, T. R., McEnaney, J. M. & Jaramillo, T. F. Design and Fabrication of a Precious Metal-Free Tandem Core–Shell p+n Si/W-Doped BiVO4 Photoanode for Unassisted Water Splitting. *Advanced Energy Materials* **7**, 1701515, doi:10.1002/aenm.201701515 (2017).

20 Brillet, J. *et al.* Highly efficient water splitting by a dual-absorber tandem cell. *Nature Photonics* **6**, 824-828, doi:10.1038/nphoton.2012.265 (2012).

21 Wang, Y. *et al.* A Single-Junction Cathodic Approach for Stable Unassisted Solar Water Splitting. *Joule* **3**, 2444-2456, doi:https://doi.org/10.1016/j.joule.2019.07.022 (2019).

22 Meng, L. *et al.* Ni/Fe codoped In2S3 nanosheet arrays boost photo‐electrochemical performance of planar Si photocathodes. *Adv. Energy Mater.* **9**, 1902135 (2019).

23 Zhou, W., Niu, F., Mao, S. S. & Shen, S. Nickel complex engineered interface energetics for efficient photoelectrochemical hydrogen evolution over p-Si. *Applied Catalysis B: Environmental* **220**, 362-366 (2018).

24 Zhang, H. *et al.* Decorating mesoporous silicon with amorphous metal–phosphorous-derived nanocatalysts towards enhanced photoelectrochemical water reduction. *J. Mater. Chem. A* **4**, 14960-14967 (2016).

25 Chen, C.-J. *et al.* Wide range pH-tolerable silicon@ pyrite cobalt dichalcogenide microwire array photoelectrodes for solar hydrogen evolution. *ACS applied materials & interfaces* **8**, 5400-5407 (2016).

26 Hou, Y. *et al.* Bioinspired molecular co-catalysts bonded to a silicon photocathode for solar hydrogen evolution. *Nature materials* **10**, 434-438 (2011).

27 Zhang, X. *et al.* Amorphous MoS x Cl y electrocatalyst supported by vertical graphene for efficient electrochemical and photoelectrochemical hydrogen generation. *Energy Environ. Sci.* **8**, 862-868 (2015).

28 Li, H. *et al.* Controllable Distribution of Oxygen Vacancies in Grain Boundaries of p‐Si/TiO2 Heterojunction Photocathodes for Solar Water Splitting. *Angewandte Chemie International Edition* **60**, 4034-4037 (2021).

29 Chen, Z., Li, Y., Wang, L., Bu, Y. & Ao, J.-P. Development of a bi-compound heterogeneous cocatalyst modified p-Si photocathode for boosting the photoelectrochemical water splitting performance. *J. Mater. Chem. A* **9**, 9157-9164 (2021).

30 Chen, C.-J. *et al.* Phosphorous-doped molybdenum disulfide anchored on silicon as an efficient catalyst for photoelectrochemical hydrogen generation. *Applied Catalysis B: Environmental* **263**, 118259 (2020).

31 Benck, J. D. *et al.* Designing active and stable silicon photocathodes for solar hydrogen production using molybdenum sulfide nanomaterials. *Adv. Energy Mater.* **4**, 1400739 (2014).

32 King, L. A., Hellstern, T. R., Park, J., Sinclair, R. & Jaramillo, T. F. Highly stable molybdenum disulfide protected silicon photocathodes for photoelectrochemical water splitting. *ACS applied materials & interfaces* **9**, 36792-36798 (2017).

33 Vijselaar, W., Tiggelaar, R. M., Gardeniers, H. & Huskens, J. Efficient and stable silicon microwire photocathodes with a nickel silicide interlayer for operation in strongly alkaline solutions. *ACS energy letters* **3**, 1086-1092 (2018).

34 Ros, C. *et al.* Charge transfer characterization of ALD-grown TiO2 protective layers in silicon photocathodes. *ACS applied materials & interfaces* **9**, 17932-17941 (2017).

35 Fan, R. *et al.* Efficient and stable silicon photocathodes coated with vertically standing nano-MoS2 films for solar hydrogen production. *ACS Applied Materials & Interfaces* **9**, 6123-6129 (2017).

36 Fan, R., Dong, W., Fang, L., Zheng, F. & Shen, M. More than 10% efficiency and one-week stability of Si photocathodes for water splitting by manipulating the loading of the Pt catalyst and TiO 2 protective layer. *J. Mater. Chem. A* **5**, 18744-18751 (2017).

37 Huang, G. *et al.* Integrated MoSe2 with n+ p-Si photocathodes for solar water splitting with high efficiency and stability. *Applied Physics Letters* **112**, 013902 (2018).

38 Vanka, S. *et al.* Long-term stability studies of a semiconductor photoelectrode in three-electrode configuration. *J. Mater. Chem. A* **7**, 27612-27619 (2019).

39 Zhou, B. *et al.* Gallium nitride nanowire as a linker of molybdenum sulfides and silicon for photoelectrocatalytic water splitting. *Nat. Commun.* **9**, 1-8 (2018).

40 Higashi, M., Domen, K. & Abe, R. Highly stable water splitting on oxynitride TaON photoanode system under visible light irradiation. *J Am Chem Soc* **134**, 6968-6971, doi:10.1021/ja302059g (2012).

41 Soliman, K. A. *et al.* Silver Nanoparticles-Decorated Titanium Oxynitride Nanotube Arrays for Enhanced Solar Fuel Generation. *Sci Rep* **7**, 1913, doi:10.1038/s41598-017-02124-1 (2017).

42 Kubota, J. & Domen, K. Photocatalytic Water Splitting Using Oxynitride and Nitride Semiconductor Powders for Production of Solar Hydrogen. *Interface magazine* **22**, 57-62, doi:10.1149/2.f07132if (2013).

43 Maegli, A. E. *et al.* Perovskite-Type LaTiO2N Oxynitrides for Solar Water Splitting: Influence of the Synthesis Conditions. *Energy Procedia* **22**, 61-66, doi:10.1016/j.egypro.2012.05.218 (2012).

44 Pan, C. *et al.* A complex perovskite-type oxynitride: the first photocatalyst for water splitting operable at up to 600 nm. *Angew Chem Int Ed Engl* **54**, 2955-2959, doi:10.1002/anie.201410961 (2015).

45 Iqbal, N., Khan, I., Yamani, Z. H. & Qurashi, A. Sonochemical Assisted Solvothermal Synthesis of Gallium Oxynitride Nanosheets and their Solar-Driven Photoelectrochemical Water-Splitting Applications. *Sci Rep* **6**, 32319, doi:10.1038/srep32319 (2016).
